# (*E*,*E*)-2-[3,4-Bis(4-methyl­benzyl­idene)-5-oxotetra­hydro­furan-2-yl­idene]propane­dinitrile

**DOI:** 10.1107/S1600536809008526

**Published:** 2009-03-14

**Authors:** Abdullah Mohamed Asiri, Seik Weng Ng

**Affiliations:** a Chemistry Department, Faculty of Science, King Abdul Aziz University, Jeddah, Saudi Arabia; b Department of Chemistry, University of Malaya, 50603 Kuala Lumpur, Malaysia

## Abstract

In the title mol­ecule, C_23_H_16_N_2_O_2_, the two exocyclic C=C bonds bearing the tolyl groups have an *E* configuration and the beznene rings are oriented at 22.1 (1) and 24.8 (1)° with respect to the mean plane of the atoms of the furan ring.

## Related literature

The compound belongs to a class of photochromic fulgicides; for similar structures, see: Asiri *et al.* (2000 ▶); Heller *et al.* (1994 ▶); Liang *et al.* (2003 ▶).
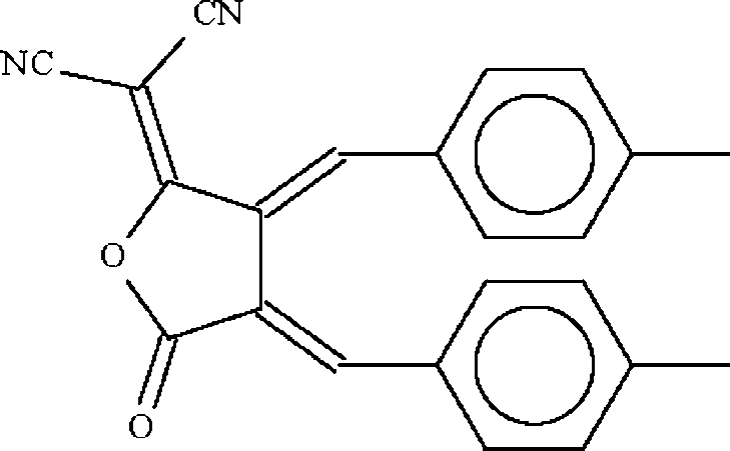


## Experimental

### 

#### Crystal data

C_23_H_16_N_2_O_2_
*M* *_r_* = 352.38
Monoclinic, 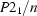

*a* = 6.7908 (2) Å
*b* = 22.0814 (5) Å
*c* = 11.8626 (3) Å
β = 96.877 (2)°
*V* = 1766.00 (8) Å^3^
*Z* = 4
Mo *K*α radiation
μ = 0.09 mm^−1^
*T* = 123 K
0.22 × 0.12 × 0.08 mm

#### Data collection

Bruker SMART APEX diffractometer
Absorption correction: none
16672 measured reflections
4051 independent reflections
2971 reflections with *I* > 2σ(*I*)
*R*_int_ = 0.040

#### Refinement

*R*[*F* ^2^ > 2σ(*F* ^2^)] = 0.044
*wR*(*F* ^2^) = 0.119
*S* = 1.02
4051 reflections
246 parameters
H-atom parameters constrained
Δρ_max_ = 0.29 e Å^−3^
Δρ_min_ = −0.24 e Å^−3^

### 

Data collection: *APEX2* (Bruker, 2008 ▶); cell refinement: *SAINT* (Bruker, 2008 ▶); data reduction: *SAINT*; program(s) used to solve structure: *SHELXS97* (Sheldrick, 2008 ▶); program(s) used to refine structure: *SHELXL97* (Sheldrick, 2008 ▶); molecular graphics: *X-SEED* (Barbour, 2001 ▶); software used to prepare material for publication: *publCIF* (Westrip, 2009 ▶).

## Supplementary Material

Crystal structure: contains datablocks global, I. DOI: 10.1107/S1600536809008526/lh2785sup1.cif

Structure factors: contains datablocks I. DOI: 10.1107/S1600536809008526/lh2785Isup2.hkl

Additional supplementary materials:  crystallographic information; 3D view; checkCIF report

## Figures and Tables

**Fig. 1.**
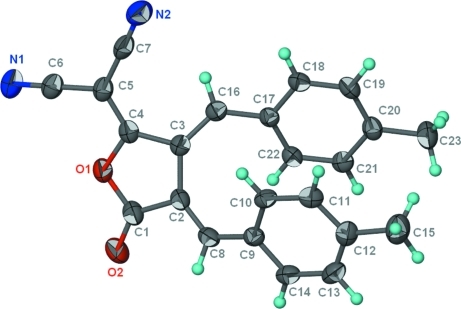
Thermal ellisoid plot (Barbour, 2001) of C23H16N2O2; probability levels are set at 70% and H-atoms are drawn as spheres of arbitrary radius.

## supplementary crystallographic information

### Comment

The molecular structure of the title compound is shown in Fig. 1.

### Experimental

Diethylamine(0.73 g,10.0 mmol) was added dropwise *E*,*E*-3,4-bis(4-tolylmethylene) succinic anhydride (1.52 g, 5.0 mmol) and malononitrile (0.33 g, 5.0 mmol) in THF (20 ml) at 273 K. The mixture was kept at this temperature for 6 h. Diethyl ether (15 ml) was added to quench the reaction. The product was dissolved in dichloromethane (20 ml) and cyclized by acetyl chloride (10 ml) at 293 K. The reaction was kept at this temperature for 10 h. The solvent and excess acetyl chloride were removed in vacuum, and the residual was chromatographed on silica gel. Elution by a 3:7 mixture of ethyl acetate and light petroleum give the title compound as an orange compound (1.41 g, 80% yield), m.p. 478–479 K. Crystals were grown with ether/lightpetroleum ether as solvent.

### Refinement

Carbon-bound H-atoms were placed in calculated positions (C—H 0.95 to 0.98 Å) and were included in the refinement in the riding model approximation, with *U*(H) set to 1.2 to 1.5*U*(C).

### Crystal data

**Table:**

C_23_H_16_N_2_O_2_	*F*(000) = 736
*M**_r_* = 352.38	*D*_x_ = 1.325 Mg m^−^^3^
Monoclinic, *P*2_1_/*n*	Mo *K*α radiation, λ = 0.71073 Å
Hall symbol: -P 2yn	Cell parameters from 2995 reflections
*a* = 6.7908 (2) Å	θ = 2.5–25.4°
*b* = 22.0814 (5) Å	µ = 0.09 mm^−^^1^
*c* = 11.8626 (3) Å	*T* = 123 K
β = 96.877 (2)°	Prism, orange
*V* = 1766.00 (8) Å^3^	0.22 × 0.12 × 0.08 mm
*Z* = 4	

### Data collection

**Table:**

Bruker SMART APEX diffractometer	2971 reflections with *I* > 2σ(*I*)
Radiation source: fine-focus sealed tube	*R*_int_ = 0.040
graphite	θ_max_ = 27.5°, θ_min_ = 1.8°
ω scans	*h* = −8→8
16672 measured reflections	*k* = −28→28
4051 independent reflections	*l* = −15→15

### Refinement

**Table:**

Refinement on *F*^2^	Primary atom site location: structure-invariant direct methods
Least-squares matrix: full	Secondary atom site location: difference Fourier map
*R*[*F*^2^ > 2σ(*F*^2^)] = 0.044	Hydrogen site location: inferred from neighbouring sites
*wR*(*F*^2^) = 0.119	H-atom parameters constrained
*S* = 1.02	*w* = 1/[σ^2^(*F*_o_^2^) + (0.0555*P*)^2^ + 0.4567*P*] where *P* = (*F*_o_^2^ + 2*F*_c_^2^)/3
4051 reflections	(Δ/σ)_max_ = 0.001
246 parameters	Δρ_max_ = 0.29 e Å^−^^3^
0 restraints	Δρ_min_ = −0.24 e Å^−^^3^

### Fractional atomic coordinates and isotropic or equivalent isotropic displacement parameters (Å^2^)

**Table:**

	*x*	*y*	*z*	*U*_iso_*/*U*_eq_	
O1	0.81235 (17)	0.53159 (5)	0.60804 (9)	0.0311 (3)	
O2	0.8934 (2)	0.56246 (6)	0.78870 (10)	0.0434 (3)	
N1	0.7342 (2)	0.40469 (7)	0.44380 (13)	0.0404 (4)	
N2	0.5427 (3)	0.55698 (7)	0.22059 (13)	0.0419 (4)	
C1	0.8373 (2)	0.57691 (7)	0.69372 (14)	0.0311 (4)	
C2	0.7782 (2)	0.63512 (7)	0.64023 (13)	0.0255 (3)	
C3	0.7583 (2)	0.62428 (7)	0.51673 (13)	0.0239 (3)	
C4	0.7526 (2)	0.55846 (7)	0.50622 (13)	0.0257 (3)	
C5	0.6993 (2)	0.52050 (7)	0.41733 (14)	0.0279 (3)	
C6	0.7187 (2)	0.45625 (7)	0.43229 (14)	0.0309 (4)	
C7	0.6140 (3)	0.54142 (7)	0.30845 (15)	0.0310 (4)	
C8	0.7414 (2)	0.68084 (7)	0.71076 (13)	0.0269 (3)	
H8	0.7747	0.6724	0.7892	0.032*	
C9	0.6596 (2)	0.74052 (7)	0.68603 (12)	0.0242 (3)	
C10	0.5317 (2)	0.75352 (7)	0.58741 (12)	0.0250 (3)	
H10	0.4849	0.7216	0.5374	0.030*	
C11	0.4733 (2)	0.81213 (7)	0.56248 (13)	0.0276 (3)	
H11	0.3863	0.8201	0.4953	0.033*	
C12	0.5393 (2)	0.86024 (7)	0.63392 (14)	0.0306 (4)	
C13	0.6552 (2)	0.84664 (8)	0.73535 (14)	0.0317 (4)	
H13	0.6955	0.8783	0.7873	0.038*	
C14	0.7129 (2)	0.78790 (7)	0.76190 (13)	0.0283 (4)	
H14	0.7897	0.7795	0.8325	0.034*	
C15	0.4873 (3)	0.92448 (8)	0.60006 (17)	0.0445 (5)	
H15A	0.5459	0.9521	0.6595	0.067*	
H15B	0.3428	0.9293	0.5902	0.067*	
H15C	0.5394	0.9339	0.5285	0.067*	
C16	0.7738 (2)	0.66042 (7)	0.42565 (13)	0.0246 (3)	
H16	0.7519	0.6408	0.3540	0.030*	
C17	0.8187 (2)	0.72444 (7)	0.42155 (12)	0.0223 (3)	
C18	0.7433 (2)	0.75755 (7)	0.32513 (12)	0.0252 (3)	
H18	0.6720	0.7372	0.2624	0.030*	
C19	0.7713 (2)	0.81920 (7)	0.32030 (13)	0.0274 (3)	
H19	0.7157	0.8410	0.2551	0.033*	
C20	0.8803 (2)	0.85031 (7)	0.40968 (13)	0.0273 (3)	
C21	0.9648 (2)	0.81676 (7)	0.50266 (13)	0.0261 (3)	
H21	1.0441	0.8367	0.5629	0.031*	
C22	0.9353 (2)	0.75505 (7)	0.50862 (12)	0.0242 (3)	
H22	0.9949	0.7331	0.5727	0.029*	
C23	0.9098 (3)	0.91778 (8)	0.40462 (16)	0.0382 (4)	
H23A	0.9312	0.9342	0.4819	0.057*	
H23B	0.7918	0.9365	0.3632	0.057*	
H23C	1.0257	0.9266	0.3654	0.057*	

### Atomic displacement parameters (Å^2^)

**Table:**

	*U*^11^	*U*^22^	*U*^33^	*U*^12^	*U*^13^	*U*^23^
O1	0.0370 (7)	0.0231 (6)	0.0326 (6)	0.0003 (5)	0.0017 (5)	0.0047 (5)
O2	0.0597 (9)	0.0357 (7)	0.0323 (7)	−0.0004 (6)	−0.0051 (6)	0.0116 (5)
N1	0.0444 (9)	0.0266 (8)	0.0501 (10)	0.0001 (6)	0.0057 (8)	−0.0024 (7)
N2	0.0523 (10)	0.0350 (8)	0.0375 (9)	0.0054 (7)	0.0009 (7)	−0.0061 (7)
C1	0.0314 (9)	0.0286 (9)	0.0327 (9)	−0.0034 (7)	0.0014 (7)	0.0057 (7)
C2	0.0258 (8)	0.0245 (8)	0.0256 (8)	−0.0029 (6)	0.0010 (6)	0.0040 (6)
C3	0.0219 (7)	0.0238 (8)	0.0261 (8)	−0.0003 (6)	0.0034 (6)	−0.0005 (6)
C4	0.0229 (8)	0.0246 (8)	0.0300 (8)	0.0009 (6)	0.0051 (6)	0.0029 (6)
C5	0.0262 (8)	0.0228 (8)	0.0355 (9)	0.0012 (6)	0.0068 (7)	−0.0006 (6)
C6	0.0276 (9)	0.0271 (9)	0.0384 (9)	0.0003 (6)	0.0054 (7)	−0.0031 (7)
C7	0.0345 (9)	0.0215 (8)	0.0378 (10)	0.0016 (6)	0.0076 (8)	−0.0067 (7)
C8	0.0284 (8)	0.0313 (9)	0.0208 (7)	−0.0053 (6)	0.0023 (6)	0.0033 (6)
C9	0.0237 (8)	0.0274 (8)	0.0224 (7)	−0.0032 (6)	0.0066 (6)	−0.0014 (6)
C10	0.0230 (8)	0.0295 (8)	0.0233 (7)	−0.0020 (6)	0.0059 (6)	−0.0060 (6)
C11	0.0229 (8)	0.0338 (9)	0.0262 (8)	0.0029 (6)	0.0038 (6)	−0.0012 (6)
C12	0.0280 (8)	0.0277 (9)	0.0384 (9)	0.0007 (6)	0.0132 (7)	−0.0027 (7)
C13	0.0314 (9)	0.0323 (9)	0.0327 (9)	−0.0066 (7)	0.0094 (7)	−0.0113 (7)
C14	0.0269 (8)	0.0356 (9)	0.0227 (8)	−0.0043 (7)	0.0047 (6)	−0.0046 (6)
C15	0.0500 (12)	0.0306 (10)	0.0552 (12)	0.0064 (8)	0.0152 (10)	−0.0012 (8)
C16	0.0229 (8)	0.0260 (8)	0.0252 (7)	0.0011 (6)	0.0044 (6)	−0.0022 (6)
C17	0.0215 (7)	0.0237 (8)	0.0226 (7)	0.0008 (6)	0.0067 (6)	0.0011 (6)
C18	0.0251 (8)	0.0298 (8)	0.0212 (7)	−0.0005 (6)	0.0046 (6)	0.0005 (6)
C19	0.0287 (8)	0.0284 (8)	0.0255 (8)	0.0030 (6)	0.0047 (6)	0.0071 (6)
C20	0.0254 (8)	0.0256 (8)	0.0323 (8)	−0.0014 (6)	0.0091 (7)	0.0035 (6)
C21	0.0229 (8)	0.0295 (8)	0.0264 (8)	−0.0040 (6)	0.0046 (6)	−0.0012 (6)
C22	0.0225 (8)	0.0269 (8)	0.0233 (7)	0.0000 (6)	0.0033 (6)	0.0035 (6)
C23	0.0431 (10)	0.0278 (9)	0.0436 (10)	−0.0045 (7)	0.0055 (8)	0.0057 (8)

### Geometric parameters (Å, °)

**Table:**

O1—C4	1.3631 (19)	C13—C14	1.381 (2)
O1—C1	1.422 (2)	C13—H13	0.9500
O2—C1	1.1890 (19)	C14—H14	0.9500
N1—C6	1.150 (2)	C15—H15A	0.9800
N2—C7	1.148 (2)	C15—H15B	0.9800
C1—C2	1.468 (2)	C15—H15C	0.9800
C2—C8	1.353 (2)	C16—C17	1.448 (2)
C2—C3	1.475 (2)	C16—H16	0.9500
C3—C16	1.357 (2)	C17—C22	1.398 (2)
C3—C4	1.459 (2)	C17—C18	1.402 (2)
C4—C5	1.361 (2)	C18—C19	1.377 (2)
C5—C7	1.428 (2)	C18—H18	0.9500
C5—C6	1.434 (2)	C19—C20	1.399 (2)
C8—C9	1.446 (2)	C19—H19	0.9500
C8—H8	0.9500	C20—C21	1.394 (2)
C9—C14	1.399 (2)	C20—C23	1.505 (2)
C9—C10	1.401 (2)	C21—C22	1.380 (2)
C10—C11	1.375 (2)	C21—H21	0.9500
C10—H10	0.9500	C22—H22	0.9500
C11—C12	1.399 (2)	C23—H23A	0.9800
C11—H11	0.9500	C23—H23B	0.9800
C12—C13	1.390 (2)	C23—H23C	0.9800
C12—C15	1.505 (2)		
			
C4—O1—C1	108.93 (12)	C13—C14—C9	120.75 (15)
O2—C1—O1	119.06 (15)	C13—C14—H14	119.6
O2—C1—C2	133.21 (16)	C9—C14—H14	119.6
O1—C1—C2	107.71 (13)	C12—C15—H15A	109.5
C8—C2—C1	116.55 (14)	C12—C15—H15B	109.5
C8—C2—C3	137.18 (14)	H15A—C15—H15B	109.5
C1—C2—C3	105.97 (13)	C12—C15—H15C	109.5
C16—C3—C4	121.42 (14)	H15A—C15—H15C	109.5
C16—C3—C2	133.53 (14)	H15B—C15—H15C	109.5
C4—C3—C2	104.18 (12)	C3—C16—C17	129.59 (14)
C5—C4—O1	116.21 (14)	C3—C16—H16	115.2
C5—C4—C3	132.95 (15)	C17—C16—H16	115.2
O1—C4—C3	110.84 (13)	C22—C17—C18	118.03 (14)
C4—C5—C7	122.89 (15)	C22—C17—C16	123.37 (13)
C4—C5—C6	120.09 (15)	C18—C17—C16	118.59 (13)
C7—C5—C6	116.96 (14)	C19—C18—C17	120.72 (14)
N1—C6—C5	179.72 (18)	C19—C18—H18	119.6
N2—C7—C5	178.35 (18)	C17—C18—H18	119.6
C2—C8—C9	130.47 (14)	C18—C19—C20	121.15 (14)
C2—C8—H8	114.8	C18—C19—H19	119.4
C9—C8—H8	114.8	C20—C19—H19	119.4
C14—C9—C10	118.09 (14)	C21—C20—C19	117.99 (14)
C14—C9—C8	119.16 (14)	C21—C20—C23	120.89 (15)
C10—C9—C8	122.74 (14)	C19—C20—C23	121.11 (14)
C11—C10—C9	120.42 (14)	C22—C21—C20	121.10 (14)
C11—C10—H10	119.8	C22—C21—H21	119.5
C9—C10—H10	119.8	C20—C21—H21	119.5
C10—C11—C12	121.42 (15)	C21—C22—C17	120.82 (14)
C10—C11—H11	119.3	C21—C22—H22	119.6
C12—C11—H11	119.3	C17—C22—H22	119.6
C13—C12—C11	117.88 (15)	C20—C23—H23A	109.5
C13—C12—C15	121.69 (15)	C20—C23—H23B	109.5
C11—C12—C15	120.43 (16)	H23A—C23—H23B	109.5
C14—C13—C12	121.09 (15)	C20—C23—H23C	109.5
C14—C13—H13	119.5	H23A—C23—H23C	109.5
C12—C13—H13	119.5	H23B—C23—H23C	109.5
			
C4—O1—C1—O2	−178.40 (16)	C2—C8—C9—C14	−151.98 (17)
C4—O1—C1—C2	3.07 (17)	C2—C8—C9—C10	26.5 (3)
O2—C1—C2—C8	−15.0 (3)	C14—C9—C10—C11	4.9 (2)
O1—C1—C2—C8	163.23 (14)	C8—C9—C10—C11	−173.64 (14)
O2—C1—C2—C3	170.13 (19)	C9—C10—C11—C12	0.1 (2)
O1—C1—C2—C3	−11.63 (17)	C10—C11—C12—C13	−4.4 (2)
C8—C2—C3—C16	32.8 (3)	C10—C11—C12—C15	174.86 (15)
C1—C2—C3—C16	−153.94 (17)	C11—C12—C13—C14	3.6 (2)
C8—C2—C3—C4	−158.09 (19)	C15—C12—C13—C14	−175.61 (16)
C1—C2—C3—C4	15.13 (16)	C12—C13—C14—C9	1.4 (2)
C1—O1—C4—C5	−172.45 (14)	C10—C9—C14—C13	−5.6 (2)
C1—O1—C4—C3	7.04 (16)	C8—C9—C14—C13	172.93 (14)
C16—C3—C4—C5	−23.9 (3)	C4—C3—C16—C17	−167.02 (14)
C2—C3—C4—C5	165.36 (17)	C2—C3—C16—C17	0.5 (3)
C16—C3—C4—O1	156.70 (14)	C3—C16—C17—C22	28.9 (2)
C2—C3—C4—O1	−14.02 (16)	C3—C16—C17—C18	−151.66 (16)
O1—C4—C5—C7	173.23 (14)	C22—C17—C18—C19	−4.7 (2)
C3—C4—C5—C7	−6.1 (3)	C16—C17—C18—C19	175.85 (14)
O1—C4—C5—C6	−3.6 (2)	C17—C18—C19—C20	1.8 (2)
C3—C4—C5—C6	177.04 (15)	C18—C19—C20—C21	1.8 (2)
C4—C5—C6—N1	−164 (100)	C18—C19—C20—C23	−179.62 (15)
C7—C5—C6—N1	19 (40)	C19—C20—C21—C22	−2.6 (2)
C4—C5—C7—N2	−147 (6)	C23—C20—C21—C22	178.83 (15)
C6—C5—C7—N2	30 (7)	C20—C21—C22—C17	−0.3 (2)
C1—C2—C8—C9	−171.89 (15)	C18—C17—C22—C21	3.9 (2)
C3—C2—C8—C9	0.8 (3)	C16—C17—C22—C21	−176.66 (14)
